# Strongly coupled Raman scattering enhancement revealed by scattering-type scanning near-field optical microscopy

**DOI:** 10.1515/nanoph-2023-0016

**Published:** 2023-03-30

**Authors:** Kang Qin, Kai Liu, Sheng Peng, Zongyan Zuo, Xiao He, Jianping Ding, Yanqing Lu, Yongyuan Zhu, Xuejin Zhang

**Affiliations:** National Laboratory of Solid State Microstructures, Collaborative Innovation Center of Advanced Microstructures, Key Laboratory of Intelligent Optical Sensing and Manipulation, Jiangsu Key Laboratory of Artificial Functional Materials, College of Engineering and Applied Sciences, Nanjing University, No. 22, Hankou Road, 210093 Nanjing, P.R. China; National Laboratory of Solid State Microstructures, Collaborative Innovation Center of Advanced Microstructures, School of Physics, Nanjing University, No. 22, Hankou Road, 210093 Nanjing, P.R. China; National Laboratory of Solid State Microstructures, Collaborative Innovation Center of Advanced Microstructures, School of Electronic Science and Engineering, Nanjing University, No. 22, Hankou Road, 210093 Nanjing, P.R. China

**Keywords:** finite-size array, near-field Raman scattering, SERS, strong coupling

## Abstract

Recent advances in near-field technology with an ultrahigh spatial resolution breaking optical diffraction limit, make it possible to further identify surface-enhanced Raman scattering (SERS) enhancement theories, and to monitor the SERS substrates. Here we verify the electromagnetic enhancement mechanism for SERS with a close-up view, using scattering-type scanning near-field optical microscopy. The array of metal-insulator-metal (MIM) subwavelength structures is studied, in which the field enhancement comes from the strong coupling between gap plasmon polariton and surface plasmon polariton modes. The near-field optical measurements reveal that SERS enhancement factor (EF) varies from one MIM subwavelength unit to another in a finite array. Besides the enhancement of isolated unit, the loss exchange phenomenon in strong coupling with a large Rabi splitting can give rise to an additional enhancement of more than 2 orders of magnitude in periodic arrays and close to 3 orders of magnitude in finite arrays. The SERS EF of the array composed of only 5 units is demonstrated to yield the best SERS performance. Our near-field optical measurements show evidence that finite-size structures embodied with strong coupling effect are a key way to develop practical high-performance SERS substrates.

## 1 Introduction

Surface-enhanced Raman scattering (SERS) spectroscopy is a surface-sensitive technique to track the spectroscopic fingerprint information of analytes [1]. Traditionally, the physical mechanism of the enhancement effect of SERS is based on the amplification of local electromagnetic (EM) field with the localized surface plasmon resonances [2]. SERS systems have been predominantly based on dimer structures, i.e. nanoparticle pairs of noble metals or nanoparticle-on-mirror configurations [3–5]. The amplified signals which are usually called ‘hot spots’ occur on the surface of the nanoparticles or in the gaps of the dimer structures [6–8]. Single-molecule SERS has been detected within extremely narrow gaps [9, 10].

The SERS enhancement factor (EF) is very sensitive to the gap configurations and geometric parameters while regular arrangement of nanogaps will promote uniformity of SERS signals. At the same time, such SERS substrates support some kinds of EM modes, such as the band-edge of surface plasmon polariton (SPP) mode [11], gap plasmon polariton (GPP) mode [12], and their coupling modes. Within the coupling regime, the linewidth of resonances can be narrower, indicating lower dissipation. The coupling between dissipative-dissipative modes takes place in most practical optical systems. The Hamiltonian of the two dissipative levels (damping rates are *γ*_1_ and *γ*_2_, respectively) in a reciprocal system can be expressed as [13]

(1) $$\begin{array}{ll} H & =H_{L}+H_{\text{exchange}}\\ & =\left[\begin{array}{cc} i\gamma_{+}/2 & 0\\ 0 & i\gamma_{+}/2 \end{array}\right]+\left[\begin{array}{cc} \beta_{1}+i\gamma_{-}/2 & g^{*}\\ g & \beta_{2}-i\gamma_{-}/2 \end{array}\right], \end{array}$$

where *β*_1,2_ are the energies of two levels, *g* and g^*^ are the reciprocal coupling coefficients, *γ*_+_ = *γ*_1_ + *γ*_2_, *γ*_−_ = *γ*_1_ – *γ*_2_, *H*_*L*_ is the background damping, *H*_exchange_ is the loss exchange term. When close to the coupling region (*β*_1_ ≈ *β*_2_ = *β*_0_), the eigenvalues can then be written as

(2) $$\beta_{\pm }=\beta_{0}+i\frac{\gamma_{+}}{2}\pm \sqrt{{\left|g\right|}^{2}+{\left(i\frac{\gamma_{-}}{2}\right)}^{2}}.$$

In addition to the inevitable background damping (*γ*_+_), coupling strength also determines the system’s loss. Reducing the resonant loss can improve the EM enhancement effect of the structures [14], so the loss changing caused by mode coupling is of great significance for modulating the field enhancement effect. The higher the quality factor (*Q*-factor) and the smaller the mode volume (*V*), then the stronger the coupling. The strong coupling takes place when *Q*/√*V* overcomes a threshold [15, 16]. In the strong coupling regime, energy exchanges between the original modes, accompanying with the generation of hybrid polariton bands and Rabi splitting. In general, *V* will govern the coupling strength for plasmonic structures with obvious dissipation [17]. Compared to the SPP mode, the GPP mode has a higher spatial confinement to the EM field and thus a smaller *V*, but its *Q*-factor is rather lower [18].

In this letter, we investigate strong coupling effect between SPP and GPP modes, which combines the characteristics of high *Q*-factor and small *V*, rendering a larger SERS EF than that of single mode resonance. The comprehensive information about highly confined EM field and SERS performance are explored by adjusting geometric parameters and directly visualizing ‘hot spots’ with high spatial resolution technique [19]. The localized EM field and the SERS signal are simultaneously mapped with sub-10 nm resolution using scattering-type scanning near-field optical microscopy (*s*-SNOM) [20], in which a sharp Si tip is used as both the near-field probe and the Raman analyte. Our results demonstrate that the structures with strong coupling effect manifest superior SERS performance. Furthermore, the favorable structures for SERS applications are finite arrays with several units, rather than periodic ones. The former could provide extremely small *V*, although the latter has largest *Q*-factor.

## 2 Methods

### 2.1 Near-field optical setup

**Figure 1:**
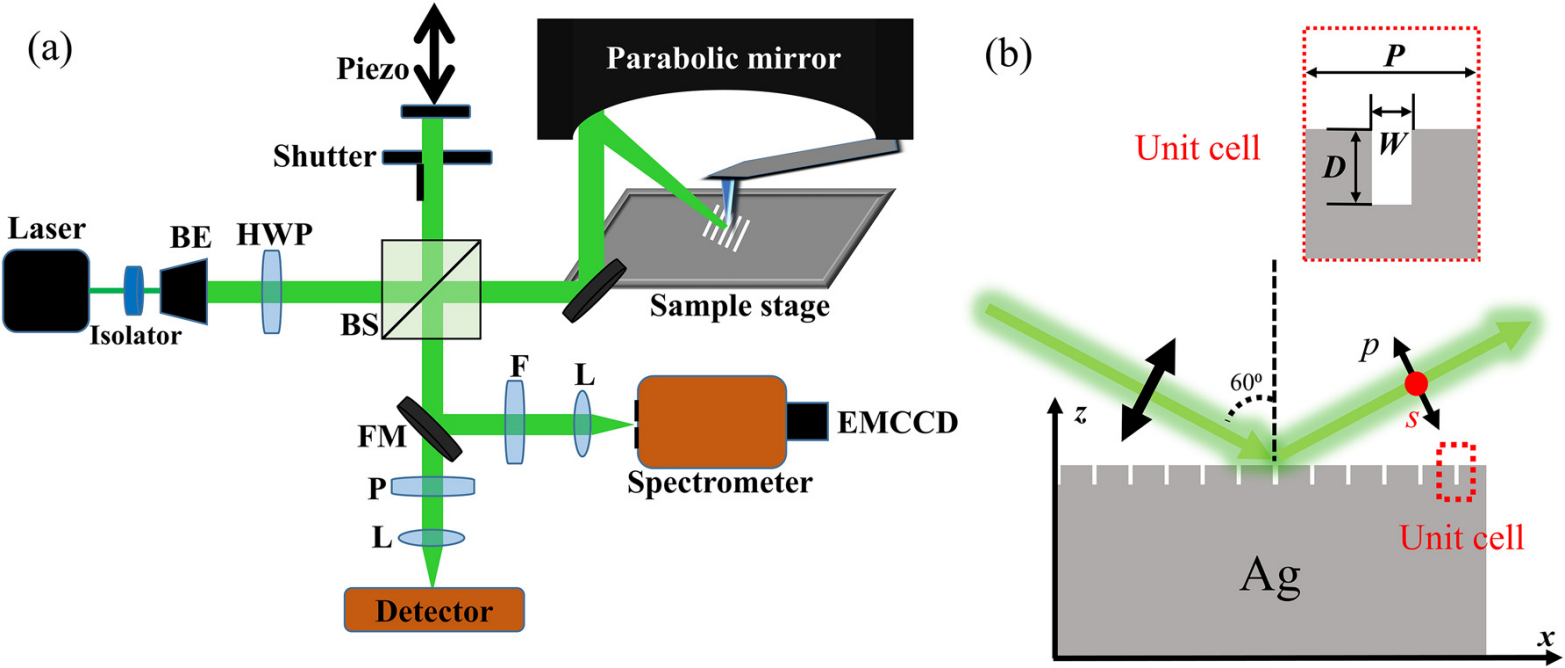
Schematics of near-field optical setup and MIM subwavelength array. (a) Scheme of the *s*-SNOM setup. The laser firstly goes through an isolator and a beam expander (BE). Then, a half-wave plate (HWP) is used to choose *s*- or *p*-polarized incident light. Parabolic mirror focuses the light onto the Si tip and collects elastic and inelastic scattered signals from the tip. Near-field signals are transmitted into the detector after a polarizer (*P*) or into the spectrometer and the EMCCD after a filter (*F*) through a flip mirror (FM). Pseudoheterodyne interferometric detection unit composed of a beam-splitter (BS) and a piezo actuator is used to record phase signals. (b) Geometry of the MIM subwavelength structure. A unit cell and geometric parameters are shown in the inset with period (*P*), width (*W*), and depth (*D*). GPP modes can be supported with appropriate *W* and *D*. The backscattered light is actually detected in the experiment.

The *s*-SNOM system with capability of collecting both near-field electric field and near-field Raman scattering signals is shown in Figure 1(a). Standard Si tip (Nanoworld, Arrow NCR) with apex diameter of less than 20 nm was used to scatter the near-field signals. Dielectric tip usually scatters purer structure signals than metal tip [21, 22]. The detected light can describe the electric field distributions, which are associated with SERS ‘hot spots’ [23]. We used a green laser (532 nm wavelength) as the source. The parabolic mirror focuses the incident laser onto the scanning Si-tip and collects the backscattered light. The tip oscillates vertically at the frequency Ω of cantilever’s mechanical resonance which is 285 kHz typically at our experiments. The background noises can be suppressed by demodulating the detected signals at a higher harmonic order of the tapping frequency, *n*Ω [24]. We collected the magnitude *s*_4_ and phase *φ*_4_ of fourth-order signals through a pseudoheterodyne Michelson interferometric detection scheme. For the inelastic Raman scattering detection, we implemented an electron-multiplying CCD (EMCCD, Princeton Instruments) and a PI (Princeton Instruments) spectrometer. A flip mirror reflects the signals into the spectrometer. And a long-pass filter blocks the elastic tip-scattered light. In order to collect different components of the electric field, a polarizer and a half-wave plate were introduced to the light path [25–27]. We study one-dimensional arrayed metal-insulator-metal (MIM) subwavelength structures, composed of grooves engraved on Ag surface, which support GPP, SPP, and their coupling modes. The narrow grooves, i.e. MIM subwavelength structures, were fabricated by focused ion beam (FIB) etching on the solution-synthesized single crystalline Ag plates [28]. The arrayed MIM subwavelength structures are illustrated in Figure 1(b). In general, the narrower the groove, the stronger the EM field for the GPP mode. Here we consider grooves with the width of 20 nm, which is accessible by common FIB etching technology, and other geometric parameters can be optimized both theoretically and experimentally (Figure S1).

### 2.2 Numerical simulations and calculations

In order to explain and verify our experimental results, we obtain the reflection spectrum from the far-field and the near-field electric field distribution results by numerical calculations. The numerical simulation software packages used were Lumerical FDTD Solutions (Lumerical, Inc.). The material properties of Ag used therein are experimental data from Johnson and Christy [29]. The *p*-polarized light impinges on the sample surface. For finite-size structures, the boundary conditions used are perfectly matched layers (PMLs), and for periodic structures, the boundary conditions are periodic boundary conditions.

## 3 Results and discussion

### 3.1 Elastic near-field optical measurements

**Figure 2:**
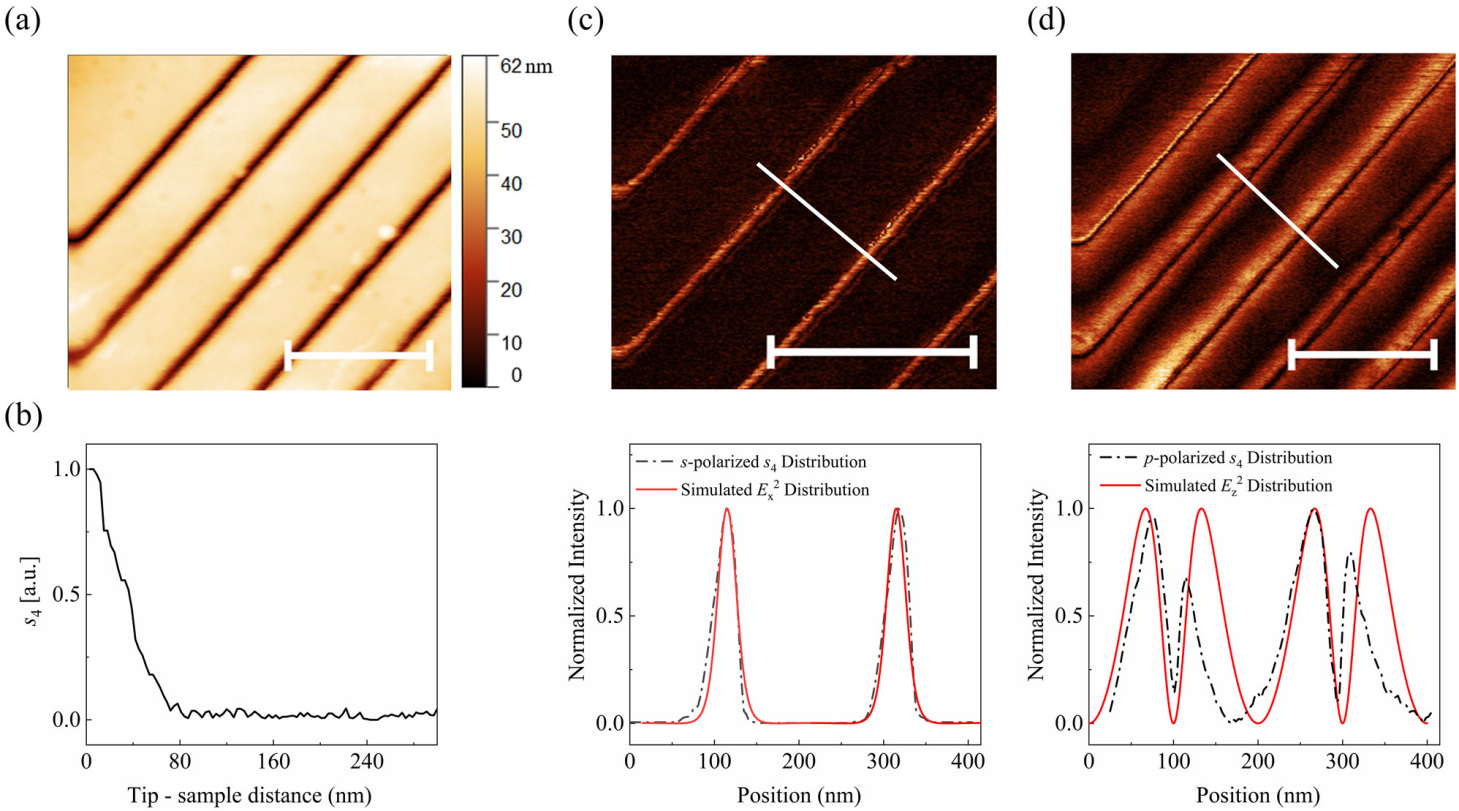
Elastic near-field optical measurements with polarization resolution. (a) Morphology of MIM subwavelength structures by AFM. (b) *s*_4_-approach curve measured on the Ag surface. (c) *s*-polarized near-field intensity mapping and line profile of *E*_*x*_^2^ component. (d) *p*-polarized near-field intensity mapping and line profile of *E*_*z*_^2^ component. The upper gives the near-field intensity distribution. The lower gives experimental and theoretical line profiles along the white line. The scale bar is 500 nm.

The acquisitions of near-field optical measurements include both intensity and phase distributions of different electric field components on the surface of structures [25–27]. The electric field within the MIM subwavelength structure is dominated by the component pointing from one metal to another metal. During near-field optical measurements, *s*- and *p*-polarized components were detected with *p*-polarized 60° incidence through adding a polarizer in front of the detector, which can be referred to Figure 1(b), but collecting the backscattered field. Under this circumstance, *s*-polarized *s*_4_ signal reflects well the in-plane component *E*_*x*_^2^ while *p*-polarized *s*_4_ signal the out-of-plane component *E*_*z*_^2^. Figure 2(a) is the surface morphology measured by AFM. Figure 2(b) shows *s*_4_ signal as a function of the tip-Ag surface distance, in which the 1/*e* decay behavior conforms the pure near-field feature of the acquired *s*_4_ signal. When the number of units *N* is large enough, the property of finite MIM subwavelength array is close to that of infinite periodic one. To investigate the periodic array, we kept *N* > 100 in the experiments, took the isolated MIM subwavelength structure with the width of 20 nm and depth of 35 nm, and changed the array period. For the MIM subwavelength structure with the period of 200 nm, the measured near-field distributions of *s*-polarized and *p*-polarized *s*_4_ components are shown in Figure 2(c) and (d) respectively. It can be seen that the strongest *s*-polarized *s*_4_ component is concentrated on the inner dielectric domain of the MIM subwavelength structure, while the strongest *p*-polarized *s*_4_ component is distributed on both sides of the MIM subwavelength structure, which correspond to the behavior of *E*_*x*_^2^ and *E*_*z*_^2^ components. To match those in experiments, the calculated results shown in Figure 2(c) and (d), as well as in the rest of the text, are represented as the near-field signals extracted 20 nm above the sample surface unless other-wise specialized. The asymmetry in measured *p*-polarized *s*_4_ distribution comes from the oblique incidence of laser (Figure S2). Above results show that the MIM subwavelength structure can generate remarkable localized field enhancement.

### 3.2 Near-field Raman scattering measurements

**Figure 3:**
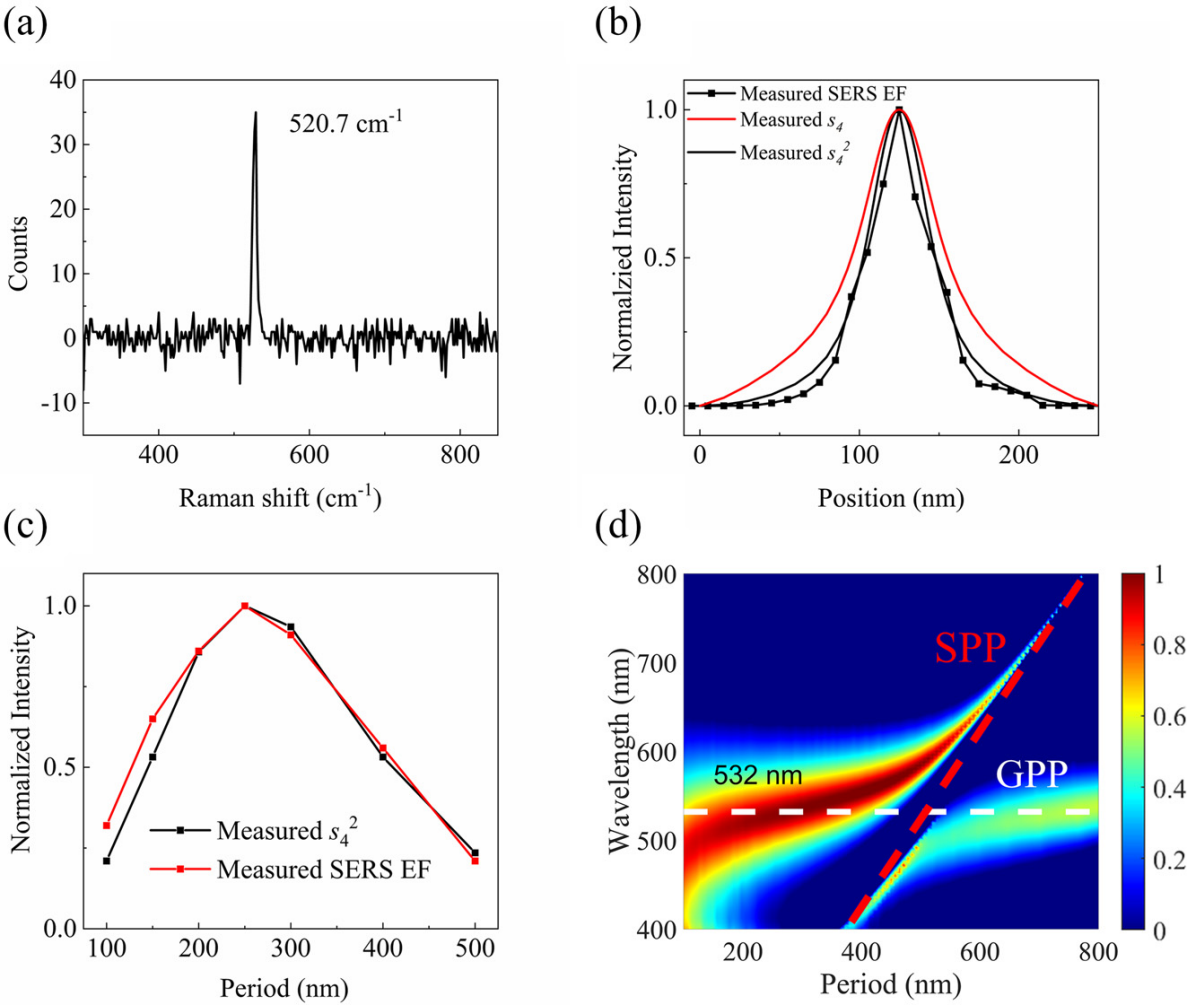
Near-field Raman scattering measurements and strong coupling mechanism. (a) Detected near-field Raman scattering signal of Si tip. (b) Normalized line profile of near-field Raman scattering intensity, elastically scattered *s*_4_, and *s*_4_^2^ along *x*-axis. (c) Period-dependent near-field Raman scattering intensity and elastically scattered *s*_4_^2^ in the center of the MIM subwavelength structure. (d) The strong coupling between GPP and SPP modes. The color represents the maximum SERS EF of MIM subwavelength unit.

The SERS EF can be defined as EF = | ***E***_e_|^4^/| ***E***_0_|^4^, where ***E***_0_ is the incident electric field and ***E***_e_ is the enhanced electric field. The total EF can be scaled as *F*^2^ = *F*_1_ × *F*_2_, where *F*_1_ is the EF of isolated MIM subwavelength structure and *F*_2_ is the additional EF that related to coupling interaction. The Stokes shifted Raman scattering spectra can be detected when the scattered near-field signals are transmitted from the tip to the EMCCD via a long pass filter. Figure 3(a) shows a typical near-field Raman scattering spectrum measured from Si tip. We scanned the Si tip along the direction perpendicular to the grooves (*x*-axis) and collected the spectra at each position with a 10 nm interval. By this way, we obtained the near-field intensity distribution of Raman scattering signals of the samples. Figure 3(b) shows the near-field intensity distribution of Si Raman scattering signal within one period, in which the curve of measured SERS EF is closer to the curve of *s*_4_^2^ rather than *s*_4_.

### 3.3 Strong coupling phenomenon

The maximum SERS EF takes place around the period of 250 nm, and the SERS EF curve matches well with the curve of *s*_4_^2^, shown in Figure 3(c). As the period becomes smaller, the coupling between adjacent GPP modes will be stronger, which results in the blueshift of the resonant wavelength of GPP modes [30]. When the period becomes so small as to be close to the mean free path of electron gas, the probability of collisions will increase and spatial non-locality emerges [31, 32], causing additional losses and leading the SERS EF to drop. The case of isolated GPP mode is close to that of very large period. Within above two limits, SPP modes are involved, altering original resonant wavelength of GPP modes. The mode coupling process can be described by coupled mode theory. With the increase of the ratio of coupling strength to the difference of damping rates, the coupling type turns from weak coupling to strong coupling. From Equation (2), reversible exchange of energy, i.e. Rabi oscillation arises between the modes, and the system’s damping rates will degenerate into the average damping rate in the strong coupling regime (Section 3 in Supporting Information). Figure 3(d) shows a significant anticrossing behavior between GPP and SPP modes, forming two new hybrid polariton bands with a Rabi splitting energy of 408.5 meV, which indicates a strong coupling phenomenon (Figure S3) [33, 34]. From Figure 3(d), the loss-exchange provides an additional enhancement of *F*_2_ ∼ 380 at the period of 250 nm by comparing with the EF at a very large period. Such enhancement benefits from the relative low damping rate of SPP mode. In addition, the SERS EF is also very sensitive to the width of MIM subwavelength structure (Figure S4).

### 3.4 Finite-size effects

**Figure 4:**
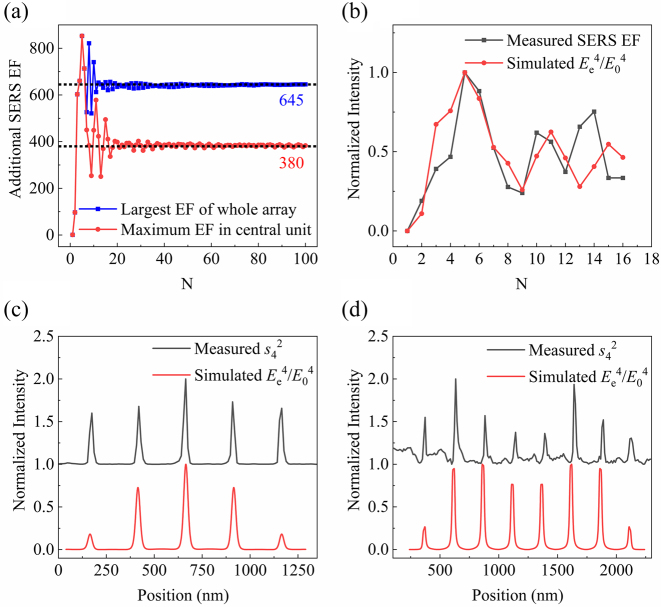
Finite-size effects. (a) Calculated SERS EF with *N*. (b) Measured and calculated maximum SERS EF of central unit with *N*. (c) Measured and calculated near-field distribution along the *x*-axis of an array with 5 MIM subwavelength units. (d) Measured and calculated near-field distribution along the *x*-axis of an array with 8 MIM subwavelength units.

For finite-size array structures, additional enhancement *F*_2_ changes with *N* and varies from one MIM subwavelength unit to another. Figure 4(a) shows the calculated maximum SERS EF on surface with *N* for both the central unit and whole array, which reveals an oscillation feature. Here the geometric parameters of the MIM subwavelength unit are fixed as 20 nm in width, 35 nm in depth and 250 nm in spacing. Most notably, the apparent oscillations for small *N* produce large additional enhancement *F*_2_. The oscillation of both tends to be stable when *N* become large, so that a finite array of MIM subwavelength units can be practically regarded as a periodic one as long as *N* > 20. When *N* > 10, oscillation period of *N* is typically around 4. The additional enhancement *F*_2_ of maximum SERS EF are around 380 for central unit and 645 for whole array respectively. The difference between above two values can be ascribed to edge effect, in which the edge provides a channel for energy leakage, making stronger EM field [35]. It is noteworthy that the effective polarizability and resonant wavelength of the edge units are also different from those in the middle [36].

In our experiments, the maximum SERS EF of the central unit was collected for arrays with different number of units *N*, as shown in Figure 4(b). Figure 4(b) shows the agreement between experiment and calculation, which can be further confirmed (Figure S5). It can be seen that the maximum SERS EF of both reaches its maximum when *N* = 5, whose near-field spatial distribution is shown in Figure 4(c). When *N* ≤ 5, the largest SERS EF appears in the central unit, which is more than 800 times than that of an isolated one. From Figure 4(b), the EF of middle unit reaches valley point when *N* = 8, whose near-field spatial distribution is shown in Figure 4(d). In this case, the largest EF does not exist in the middle unit, but move towards the boundary units.

### 3.5 EM confinement in time and in space

**Figure 5:**
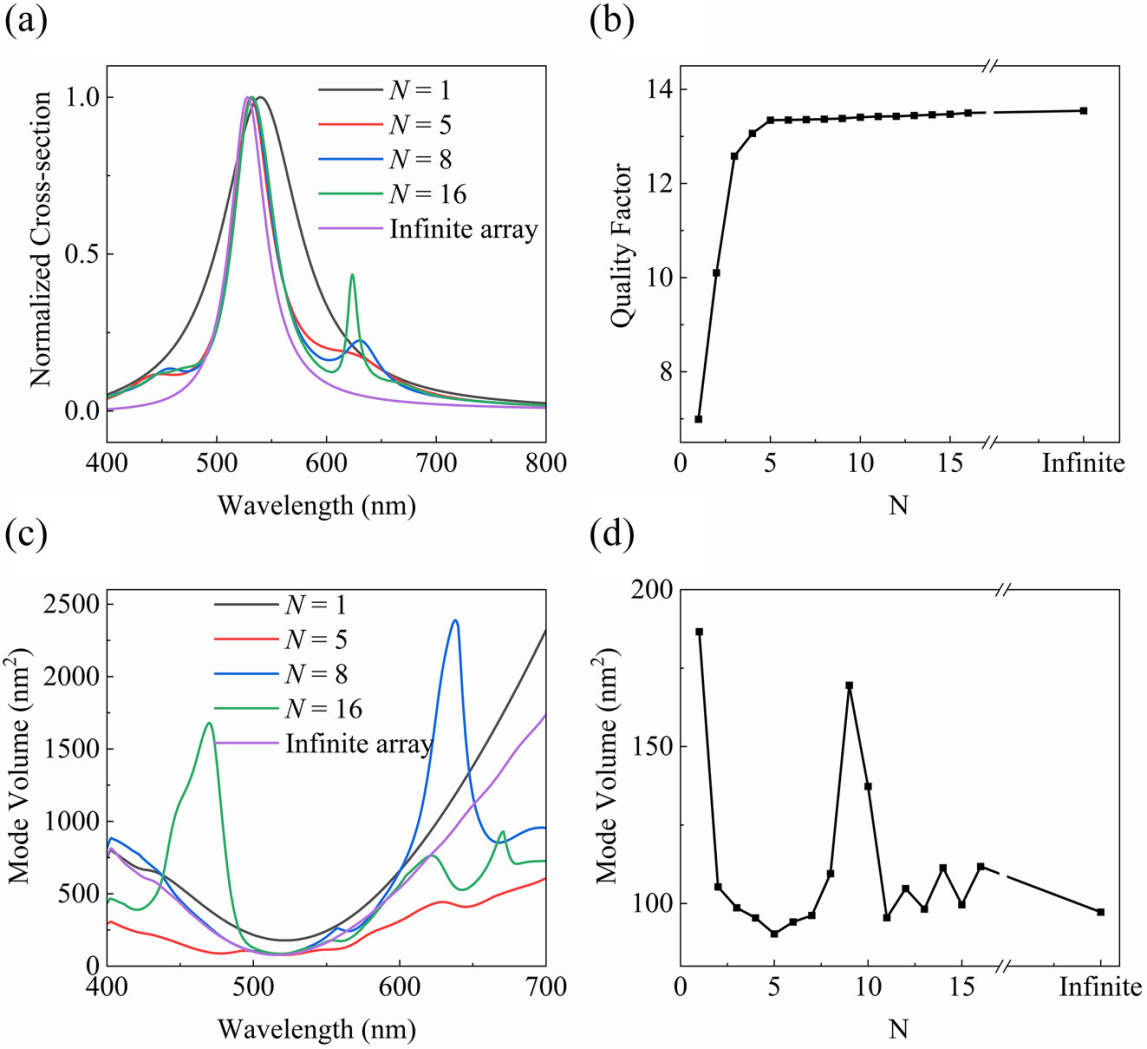
EM confinement in time and in space. (a) Calculated scattering cross section with *N*. (b) Calculated *Q*-factor with *N* at 532 nm. (c) Calculated *V* with wavelength and *N*. (d) Calculated *V* with *N* at 532 nm. The geometric parameters of MIM subwavelength unit are 20 nm in width, 35 nm in depth, and 250 nm in spacing respectively.

The strong coupling between GPP and SPP modes accounts for localized near-field enhancement by confining EM waves in space and in time. The confinement characteristics of cavity quantum electrodynamics provide valuable insights into the light–matter interaction process of the SERS. The *Q*-factor governs the temporal confinement while *V* the spatial confinement. *Q*-factor represents the damping rate of the resonance mode, and is an important indicator in non-Hermitian physical processes [13, 37, 38]. For MIM subwavelength structures, we calculated scattering cross section of the central unit for different numbers of units *N* [37, 38], as shown in Figure 5(a). It shows that prominent resonance peaks near 532 nm and the resonance blueshifts with *N*, which is similar to the case of reducing the period shown in Figure 3(d). Figure 5(b) show the *Q*-factor extracted from the scattering cross section varies with *N*. For *N* ≤ 5, the *Q*-factor increases abruptly with *N*. When *N* = 5, the *Q*-factor is already close to that of infinite structures. Very small *V* gives birth to enormous EM field intensity and SERS EF. Figure 5(c) shows the calculated *V* of the central unit with *N* [39]. All the minimum versus situate at around 532 nm, which means that the finite MIM subwavelength arrays resonate at around 532 nm, with strong EM field confinement in space. Figure 5(d) shows the calculated *V* at 532 nm for different *N*, in which *V* oscillates with *N*. For small *N*, *V* decreases significantly with *N*, and reaches the minimum when *N* = 5. Therefore, compared to isolated MIM subwavelength structure, an array with a few MIM subwavelength units will increase *Q*-factor, reduce *V*, and thus boost the EM field confinement of the central unit, being in line with the SERS EF shown in Figure 4(b).

## 4 Conclusions

In conclusion, we have taken the GPP mode as the isolated resonance, and specially investigated the contribution of strong coupling between GPP and SPP modes in finite and infinite arrays to the SERS EF using the *s*-SNOM. The spatial distribution of SERS EF transforms with the spacing and number of MIM subwavelength units. By virtue of strong coupling, periodically arranged MIM subwavelength structures provide more than 645 times additional SERS EF compared to an isolated one. Nevertheless, it is not necessary to utilize the array with a large number of MIM subwavelength units. The additional SERS EF of the array composed of only 5 units can be more than 800. Our results demonstrate that finite arrays with strong coupling effect will facilitate the development of high-performance devices, such as SERS substrates for single-molecule detection.

## Supplementary Material

Supplementary Material Details
