# Supplementary material for: Strongly coupled Raman scattering enhancement revealed by scattering-type scanning near-field optical microscopy
Source: Nanophotonics. 2023 Mar 30;12(10):1857–64. doi: 10.1515/nanoph-2023-0016 (PMC11502024; doi:10.1515/nanoph-2023-0016)
Supplement: Supplementary file 1 — Supplementary Material Details [file j_nanoph-2023-0016_suppl_001.docx]

Supplementary Materials for “Strongly coupled Raman scattering enhancement revealed by scattering-type scanning near-field optical microscopy”

Kang Qin,^1,2,3^ Kai Liu,^1,4^ Sheng Peng,^1,4^ Zongyan Zuo,^1,5^ Xiao He,^1,4^ Jianping Ding,^1,4^ Yanqing Lu,^1,2,3^ Yongyuan Zhu,^1,4^ and Xuejin Zhang^1,2,3,^*

^1^National Laboratory of Solid State Microstructures, and Collaborative Innovation Center of Advanced Microstructures, Nanjing University, Nanjing 210093, China

^2^ Key Laboratory of Intelligent Optical Sensing and Manipulation, and Jiangsu Key Laboratory of Artificial Functional Materials, Nanjing University, Nanjing 210093, China

^3^College of Engineering and Applied Sciences, Nanjing University, Nanjing 210093, China

^4^School of Physics, Nanjing University, Nanjing 210093, China

^5^School of Electronic Science and Engineering, Nanjing University, Nanjing 210093, China

*Email: [xuejinzh@nju.edu.cn](mailto:xuejinzh@nju.edu.cn)

Section 1. Geometric parameter optimization

The MIM subwavelength structures can support high-order GPP modes.^1^ The calculated SERS EF is mapped with the depth and width at 532 nm wavelength for periodic array with the period of 250 nm, as shown in Figure S1(a). The simulation method is 3D Lumerical FDTD Solutions. A, B, and C are related to different orders of the GPP modes when the width is 20 nm. Experimentally, we used the first-order GPP mode, and investigated reflection spectra for the MIM subwavelength structures with different depth, fixed period of 250 nm, and width of 20 nm. Figure S1(b) shows the mapping image of measured reflection spectra, where every valley of the reflection spectrum for a specific depth marks the resonance of arrayed MIM subwavelength structure. Resonant wavelength redshifts with the depth. It shows that the reflectivity reaches the lowest when the depth is around 35 nm, which agrees with the calculated result in Figure S1 (a).


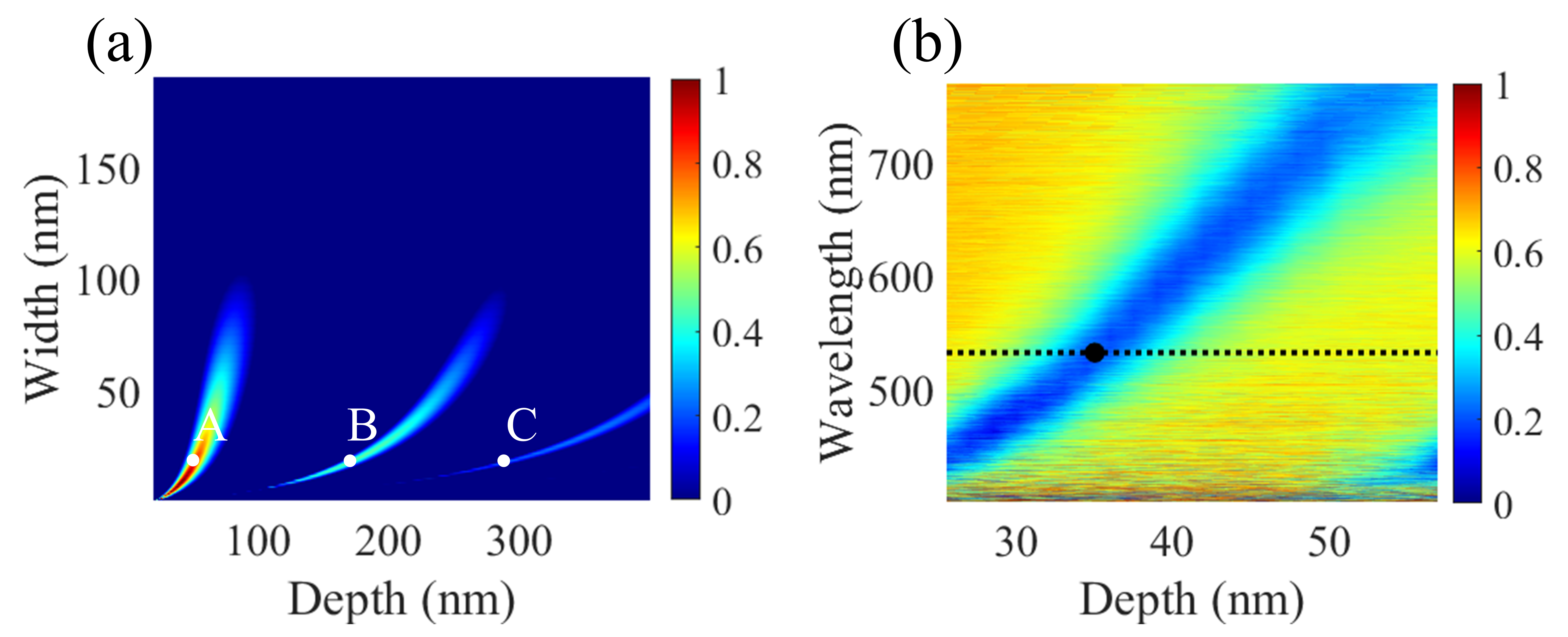


Figure S1. (a) The normalized calculated SERS EF with the depth and width at 532 nm laser excitation for periodic array with 250 nm period. A, B and C is in relation with 1^st^, 2^nd^ and 3^rd^ order GPP modes for the periodic array of MIM subwavelength unit with 20 nm width. (b) Measured reflectivity mapping with different depths and wavelengths. Black dotted line infers 532 nm wavelength. The bigger black dot indicates that the resonant wavelength at 532 nm corresponds to the MIM subwavelength structure with a depth around 35 nm.

Section 2. In-plane and out-of-plane electric field components under oblique incidence

Under *p*-polarized normal incidence, the near-field distribution of both *E_x_*^2^ and *E_z_*^2^ components along *x* direction are symmetric, and *E_x_*^2^ is about 3 times as much as *E_z_*^2^, as shown in Figure S2(a). In the *s*-SNOM measurements, the incident angle is about 60° relative to normal incidence. The detected *s*- and *p*-polarized components of *s*_4_ signal are shown in Figure S2(b). It can be seen that *s*-polarized component remains symmetric while *p*-polarized component asymmetric, and *E_x_*^2^ is only about 2 times as much as *E_z_*^2^. Figure S2(c) shows the calculated near-field distributions of *E_x_*^2^ and *E_z_*^2^ components under 60° incidence, which have some similarities and differences from those in Figures S2(a) and S2(b) in shape and strength. The asymmetry comes from the asymmetric distribution of surface charges on the two sides of the MIM subwavelength structure under oblique incidence. Figure S2(c) only partially interprets the results of Figure S2(b). In fact, the measured near-field distribution results from the complexity of multiple-scattering process of the tip and the MIM subwavelength structure.^2^


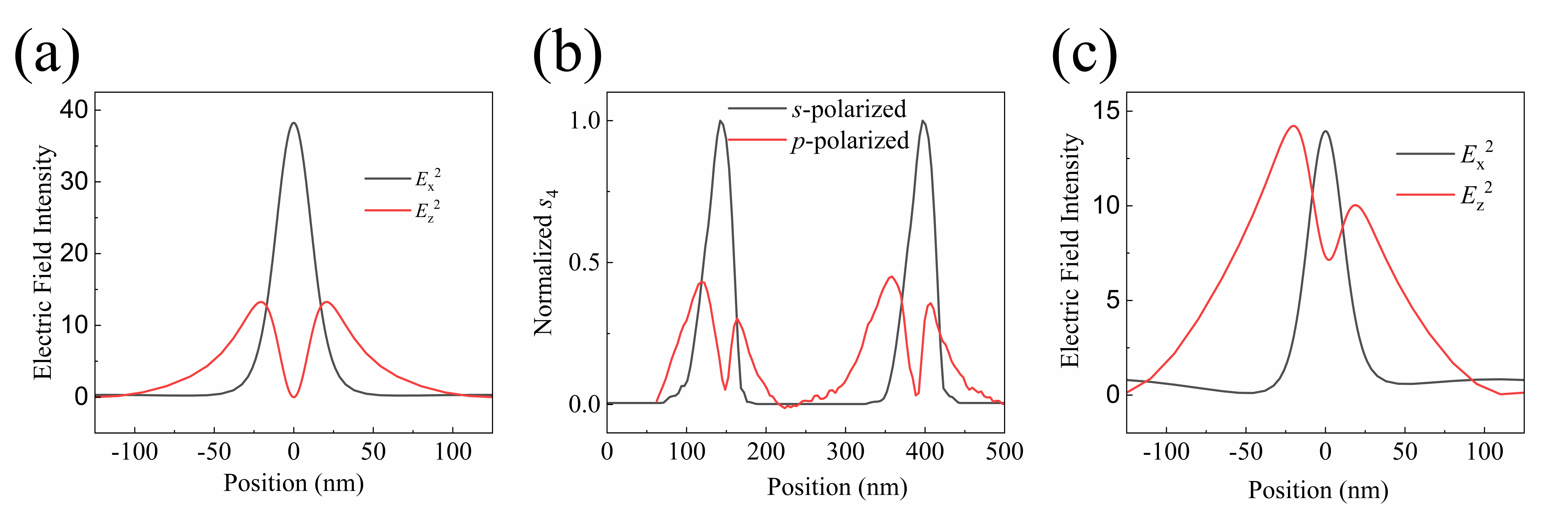


Figure S2. (a) Calculated *E_x_*^2^ and *E_z_*^2^ distributions for normal incidence. (b) Measured *s*_4_ signal using the *s*-SNOM. (c) Calculated *E_x_*^2^ and *E_z_*^2^ distributions for 60° incidence. All the above results are taken from 20 nm above the surface of MIM subwavelength structures.

Section 3. The strong coupling phenomenon

The additional enhancement is provided by the strong coupling effect between SPP and GPP modes. The system is reciprocal ($g_{12}^{*}=g_{21}=g$), thus the Hamiltonian of coupled system can be written as^3^

$H=\left[ \begin{matrix} i\gamma_{+}/2 & 0 \\ 0 & i\gamma_{+}/2 \end{matrix} \right]+\left[ \begin{matrix} E_{\mathrm{GPP}}+i\gamma_{-}/2 & g^{*} \\ g & E_{\mathrm{SPP}}-i\gamma_{-}/2 \end{matrix} \right]$.

*E*_GPP_ and *E*_SPP_ are the uncoupled resonance energy of GPP and SPP modes respectively, γ_GPP_ and γ_SPP_ are their corresponding damping rates, $\gamma_{+}=\gamma_{\mathrm{GPP}}+\gamma_{\mathrm{SPP}}$, $\gamma_{-}=\gamma_{\mathrm{GPP}}-\gamma_{\mathrm{SPP}}$, and *g* is the coupling strength. The eigenvalues can then be written as

$E_{\pm}=\frac{E_{\mathrm{GPP}}+E_{\mathrm{SPP}}+i\gamma_{+}}{2}\pm\sqrt{\left| g \right|^{2}+\frac{1}{4}\left( \delta-i\gamma_{-} \right)^{2}}$ ,

where *δ* = *E*_GPP_ – *E*_SPP_. The Rabi splitting energy, *ħΩ*, can be given when *E*_GPP_ = *E*_SPP_,

$\hbar\Omega=\sqrt{{4\left| g \right|}^{2}-\gamma_{-}^{2}}$ .

The Rabi splitting energy should be a real value in PT-symmetric phase, which corresponds to the strong coupling phenomenon, resulting in the following relation:

$$2\left| g \right|>\left| \gamma_{-} \right|=\left| \gamma_{\mathrm{GPP}}-\gamma_{\mathrm{SPP}} \right|.$$

Considering the existence of energy dissipations, *g >>* γ_GPP_, γ_SPP_ can make strong coupling be observed. A common criterion of strong coupling is^4^

$\hbar\Omega>\frac{\gamma_{\mathrm{GPP}}+\gamma_{\mathrm{SPP}}}{2}$.

The strong coupling effect can be further understood by the analysis of the reflection spectra, as shown in Figure S3. If reflection spectra in Figure S3(a) are rearranged, as shown in Figure S3(b), we can view the evolution process of strong coupling from another perspective. The two hybrid polariton bands are gradually approaching, closest at a period of 525 nm, and then moving away. The Rabi splitting energy *ħΩ* is around 408.5 meV, as shown in Figure S3(c). The damping rates γ_GPP_ and γ_SPP_ can be got from uncoupled GPP and SPP modes separately. From Figure S3(d), the damping rates of GPP and SPP modes are γ_GPP_ ~ 286.4 meV and γ_SPP_ ~ 27.4 meV respectively. It can be seen that *ħΩ* is obviously larger than γ_GPP_/2 + γ_SPP_/2, leading to a strong coupling phenomenon.^5–7^


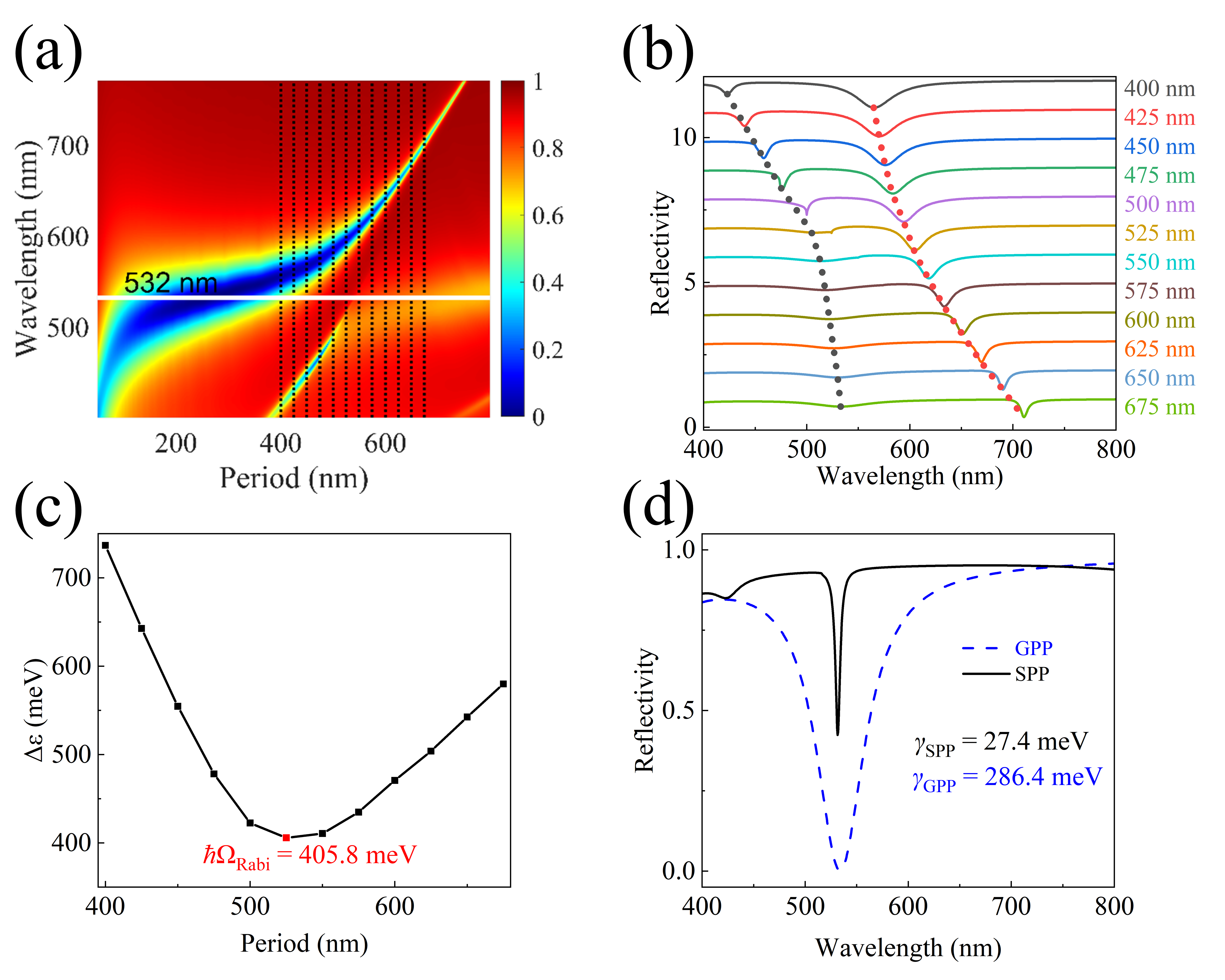


Figure S3. (a) Calculated reflectivity mapping with different periods and wavelengths for MIM subwavelength unit with 20 nm width and 35nm depth. White line refers to 532 nm in wavelength. (b) Reflection spectra extracted from vertical black dotted lines in (a). The corresponding periods are labelled on the right for each line profiles. The dotted lines are guides to the eye. (c) Energy difference of two hybrid polariton bands at different periods, the red point indicates the Rabi splitting energy is 405.8 meV. (d) Individual reflectivity for GPP and SPP modes.

Section 4. The influence of width on the near-field SERS EF

The width of MIM subwavelength structure also plays an important role in the SERS EF. Experimentally, we changed the width for a periodic array with the depth of 35 nm and period of 250 nm. Figure S4 shows that the EF decreases rapidly with the width.





Figure S4. Measured and simulated SERS EF with the width.

Section 5. Comparison between SERS EFs on and 20 nm above surface

During the *s*-SNOM measurements, *s*_4_ signal came from an average position 20 nm above the sample surface, around which tips oscillated vertically. Figure S5 shows that the measured results are in accordance with those on surface, therefore the *s*-SNOM is eligible for the near-field measurements.





Figure S5. Calculated maximum EFs of central unit on and 20 nm above sample surface.

# References

[1] W. C Tan, T. W. Preist, J. R. Sambles, and N. P. Wanstall, “Flat surface-plasmon-polariton bands and resonant optical absorption on short-pitch metal gratings,” *Phys. Rev. B*, vol. 59, no. 19, pp. 12661–12666, 1999.

[2] T. Neuman, P. Alonso-González, A. García-Etxarri, M. Schenell, R. Hillenbrand, and J. Aizpurua, “Mapping the near fields of plasmonic nanoantennas by scattering-type scanning near-field optical microscopy,” *Laser Photonics Rev*., vol. 9, no. 6, pp. 637–649, 2015.

[3] H. Zhao, L. Feng, “Parity-time symmetry photonics,” *Natl. Sci. Rev.,* vol. 5, no. 2, pp. 183–199, 2018.

[4] Y. M. Qing, Y. Z. Ren, D. Y. Lei, H. F. Ma, and T. J. Cui, “Strong coupling in two-dimensional materials-based nanostructures: a review,” *J. Opt.*, vol. 24, no. 024009, 2022.

[5] E. L. Runnerstrom, K. P. Kelley, T. G. Folland, et al., “Polaritonic Hybrid-Epsilon-near-Zero Modes: Beating the plasmonic confinement vs propagation-length trade-off with doped cadmium oxide bilayers,” *Nano Lett.*, vol. 9, pp. 948–957, 2019.

[6] L. Novotny, “Strong coupling, energy splitting, and level crossings: A classical perspective,” *Am. J. Phys.*, vol. 78, no. 11, pp. 1199–1202, 2010.

[7] P. Törmä, and W. L. Barnes, “Strong coupling between surface plasmon polaritons and emitters: a review,” *Rep. Prog. Phys.*, vol. 78, no. 013901, 2015.
